# Development and Analysis of a CNN- and Transfer-Learning-Based Classification Model for Automated Dairy Cow Feeding Behavior Recognition from Accelerometer Data

**DOI:** 10.3390/s23052611

**Published:** 2023-02-27

**Authors:** Victor Bloch, Lilli Frondelius, Claudia Arcidiacono, Massimo Mancino, Matti Pastell

**Affiliations:** 1 Natural Resources Institute Luke (Finland), Latokartanonkaari 9, 00790 Helsinki, Finland; 2 Department of Agriculture, Food and Environment (Di3A), Building and Land Engineering Section, University of Catania, Via Santa Sofia 100, 95123 Catania, Italy

**Keywords:** cow behavior, CNN classifier, acceleration tags, transfer learning, dataset variability, open-source dataset

## Abstract

Due to technological developments, wearable sensors for monitoring the behavior of farm animals have become cheaper, have a longer lifespan and are more accessible for small farms and researchers. In addition, advancements in deep machine learning methods provide new opportunities for behavior recognition. However, the combination of the new electronics and algorithms are rarely used in PLF, and their possibilities and limitations are not well-studied. In this study, a CNN-based model for the feeding behavior classification of dairy cows was trained, and the training process was analyzed considering a training dataset and the use of transfer learning. Commercial acceleration measuring tags, which were connected by BLE, were fitted to cow collars in a research barn. Based on a dataset including 33.7 cow × days (21 cows recorded during 1–3 days) of labeled data and an additional free-access dataset with similar acceleration data, a classifier with F1 = 93.9% was developed. The optimal classification window size was 90 s. In addition, the influence of the training dataset size on the classifier accuracy was analyzed for different neural networks using the transfer learning technique. While the size of the training dataset was being increased, the rate of the accuracy improvement decreased. Beginning from a specific point, the use of additional training data can be impractical. A relatively high accuracy was achieved with few training data when the classifier was trained using randomly initialized model weights, and a higher accuracy was achieved when transfer learning was used. These findings can be used for the estimation of the necessary dataset size for training neural network classifiers intended for other environments and conditions.

## 1. Introduction

Farm animal activity recognition is important for livestock health and welfare monitoring. Sensors for the behavioral recognition of dairy cows have been developed and produced for at least two decades [1]. Based on acceleration tags, numerous commercial systems [2,3,4] can provide high accuracy in behavior recognition. Nevertheless, the commercial systems usually do not provide access to the raw acceleration data, which is highly important for researchers studying the animal behavior and developing new methods for efficient farm management. In addition, the price of the equipment and its maintenance is impractical for small farms or farms with small ruminants.

New sensors are constantly being developed in this research. This has been inspired by new technologies that provide a smaller device size [5], better data transfer possibilities, and a lower energy consumption, such as Bluetooth low energy—BLE [6]. Due to progress made in data processing methods such as deep neural networks, the accuracy and robustness of the algorithms monitoring the animal behavior have been constantly improved [7]. The machine learning model development process includes data pre-processing (e.g., handling the records with missing data, filtering the raw time series, calculating additional time series, and segmenting the time series into time windows), calculating features for some classifiers, and model training and postprocessing. Riaboff et al. [7] provided an extensive review of these aspects in livestock applications.

In recent years, methods based on convolutional neural networks (CNN) have been widely used for recognition applications such as human activity recognition (HAR) [8,9]. In livestock applications, CNN for acceleration data, which is measured by tags fitted to the animals, has been used by [10,11,12]. The design of a neural network (NN)-based behavior classifier includes a number of factors. In particular, the type and the architecture of the NN must be fitted to its application. For livestock, CNNs and recurrent NNs (RNNs) with 2–4 convolutional layers have been utilized to process time series data. However, deep learning models, particularly CNNs, are still not used widely [7].

To train deep neural networks effectively, a large amount of reference data must usually be collected and labeled [13]. Different sensors are used to achieve labeled references for the cow behavior and body position: e.g., feeders for estimating the feeding time [14], or halter sensors to measure rumination and feeding time [10]. Manual labeling can be performed from direct cow observation [15] or video recorded by cameras installed in the cow environment [16]; however, this method is highly time-consuming. In cases where observations of actual behavior cannot be made, unsupervised methods for the behavior classification are used [17]. To advance the development of HAR, some researchers published data used for their studies in open access, saving time for reference preparation and enabling the use of larger datasets for model training [18] (wireless sensors data mining—WISDM). According to [7], a large variety of collected data for the classifier training have been used in different studies (from 2 to 200 h), and a recommendation to collect data from at least from 25 animals for at least 40 h was given. An analysis of required training data was performed for some data series [19]. However, no analysis of a required amount of the training data was found for farm animal activity recognition and HAR.

Different types of data augmentation have been used to enlarge the training dataset [12,20]: rotation, permutation, jittering and scaling performed for the original signal, or local averaging as a down-sampling technique and shuffling in the feature space [21]. However, the specific augmentation, as well as the optimization of the classification window length (epoch, time window, segment, observation) was performed in each study for its specific datasets.

Transfer learning is a method that prepares a classification model for one dataset and uses this pretrained model as a base for training a model for another similar dataset. For example, training a pretrained model based on younger population groups and using it as the initial condition to train a model for older people, as was carried out by [19,20]. The additional training of pretrained models using data from specific objects and environments improves their accuracy and decreases the training time relative to newly trained models or existing models. In HAR, this method was used by [22,23]. According to our review, this method has not been used for livestock activity recognition.

In this study, we evaluated the minimal amount of data needed to effectively train an NN classifier and the use of transfer learning based on an openly available dataset. We used a low-cost, open-source system based on acceleration tags to develop a behavior classification method according to the best practices taken from the reviewed studies. The aims of this study were to (a) develop a behavior classification model, (b) evaluate the impact of the size of training dataset and (c) evaluate the use of transfer learning on the accuracy of farm animal activity recognition classification using CNNs.

## 2. Materials and Methods

### 2.1. Barn Study Area and Monitored Cows

**Figure 1:**
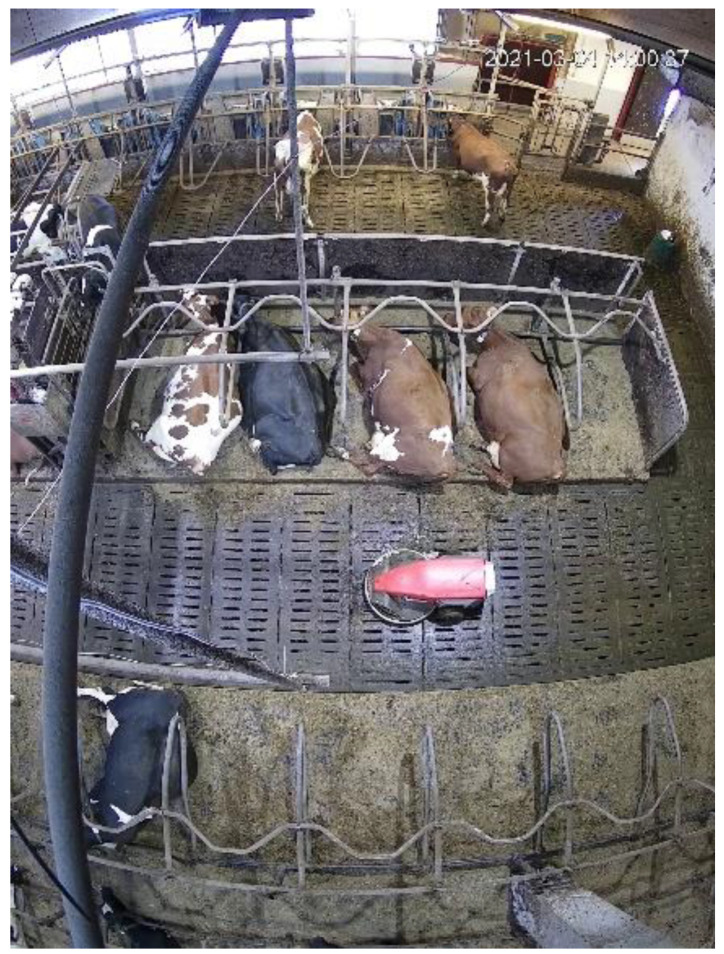
Top-view image of the research barn acquired by one of the cameras.

The data used for the development and validation of the system were collected from dairy cows housed in a free-stall research barn (Luke, Maaninka, Finland) from 4 March till 15 April 2021. The barn comprised two separated sections with a 10 × 20 m area containing, in total, 48 lying stalls and 24 feeders (Hokofarm, Marknesse, The Netherlands) as shown in Figure 1. A group of 48 cows, specifically Ayrshire (*n* = 18) and Holstein (*n* = 30) cows, were housed in the study area during the lactation period (the average parity was 2.3 with a minimum of 1 and a maximum of 7, the average ± STD days of lactation were 126 ± 82). The barn was equipped with continuously recording cameras (HDBW5541R, 5M, 3.6 mm, Dahua, Hangzhou, China). The cameras were installed on the ceiling at a height of 6 m and covered a major part of the area. Each section of the barn included a 140 m^2^ free area, winter natural ventilation, automatic manure scraping (Lely Discovery 90SW, Lely, Maassluis, The Netherlands), fresh feed delivered six times daily and freely available water.

### 2.2. System Design

**Figure 2:**
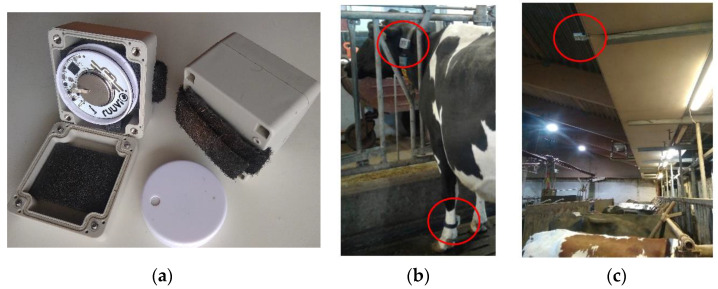
Component of the location and acceleration measuring system installed in a barn: RuuviTag inside a protecting plastic box (**a**), tag on the cow collar (**b**) and receiving station installed on a barn structure (**c**) marked by red circles.

Tags measuring 3D acceleration (RuuviTag, Ruuvi Innovations, Riihimäki, Finland) were packed in plastic boxes and adjusted by a Velcro belt on the left side of the cow collars or to one of the legs just above the metatarsal joint (Figure 2). In total, 96 tags were fitted to both the collars and legs of 48 cows. The tags attached to the legs were used only to test the reliability of the wireless transmission. The number of transmitting tags was typical for a commercial barn. The tags broadcasted the data as BLE advertising packets. The acceleration was sampled at 25 Hz, and the frequency of the message sending was 5 Hz. Each packet included five samples for three axes, which amounted to 15 acceleration values. The data from the tags were received by six receiving stations, which were single-board computers equipped with a Bluetooth antenna (Raspberry Pi 3 B+, Raspberry Pi Foundation, Cambridge, UK). The stations were packed in hermetic cases with heat sink ribs and installed at 3–5 m height on the barn structures (Figure 2c). They were evenly distributed in the study area to minimize the distance from the tags sufficient for receiving the broadcasted signal [24]. The receiving stations recorded the tag accelerations and the receiving time. The data were stored on the base station’s storage and were sent via a local network, which was maintained by a router (EA7500, Linksys), through a message-queuing protocol (ZeroMQ, iMatix Corporation, Brussels, Belgium). A PC (Intel(R) Core(TM) i7-9750H, CPU 2.6 GHz, RAM 16 GB) received the messages and stored the raw data in CSV files. The tag and base station software were written in C++ (version 2020), and the C# language was used (Microsoft, Redmond, WA, USA) for the PC. The lifetime of the tag battery was estimated using power profiling, which was described in [24] as about three years.

### 2.3. Data Collection and Labeling

Three feeding behavior classes were considered in this study: feeding, ruminating and other (neither ruminating nor feeding).

The individual feeders were used to collect reference data for the feeding. We assumed that animals were not ruminating while registered to the feeders. Manual labeling was used to recognize ruminating and other behaviors. Individual cows were recognized by their unique coat color patterns; images of cows from both sides, on top and from the head were captured at the beginning of the experiment to aid in recognition. Only time intervals during which the cow and its behavior were clearly detected were labeled. The time labels of the feeders and the cameras were synchronized with the time label of the tag receiving stations with an accuracy of ±1 s. The labeling was performed by one trained person. An ethogram of behavior classification for visual observations from [4] was used in this study.

**Figure 3:**
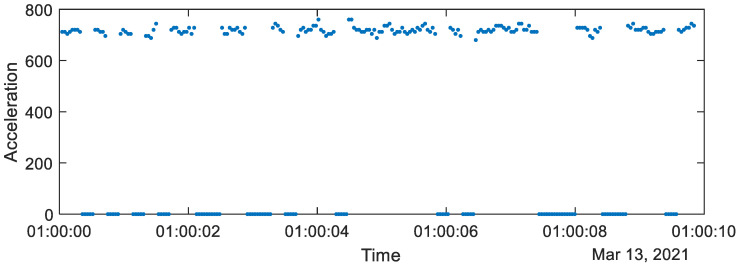
Illustration of the missing samples (value 0) in the recorded acceleration data (dots).

The average rate of the missing data messages was 52.6 ± 6.1% (mean ± STD). The missing samples were concentrated in groups containing multiplication of five samples (since the data were transferred in packets including five samples), as is shown in Figure 3.

The classification models were trained on two datasets (Table 1): data collected in this study and labeled for 21 cows, as explained above (4–13 March 2021), and open-source data published by [10]. Different from the current study, [10] used sampling at 10 Hz and sensors with the ability to download the data, thus preventing data loss. All data labeling was performed automatically with the help of halters that measured the cows’ feeding behavior (Rumiwatch, Ettenhausen, Switzerland).

### 2.4. Data Processing

Data pre-processing included filtering, amplitude normalization, sampling frequency normalization, augmentation and balancing. The raw acceleration data from the neck tags was filtered by a Hamming high-pass filter with a filter order of 511 and a cut-off frequency of 0.1 Hz. The acceleration values were normalized to ±1. Due to a high rate of missing samples, the missing data were replaced by zeros to preserve the structure of the time series. The data were used for the training according to the methods proposed by [25].

A window overlap augmentation of consecutive windows with a 50% overlap was used to increase the amount of the training data in accordance with the recommendations of [7] and the review by [8]. Since tags fitted on collars were able to rotate around the neck, a rotational augmentation was used to simulate a possible rotation around the X axis parallel to the cows’ neck and to train the model to be insensitive to the tag orientation. The Y and Z acceleration components were rotated in a 3D space by the transformation

(1) $$\left(\begin{array}{c} a_{X}\\ a_{Y}\\ a_{Z} \end{array}\right)=\left(\begin{array}{ccc} 1 & 0 & 0\\ 0 & cos\alpha & -sin\alpha \\ 0 & sin\alpha & cos\alpha \end{array}\right)\left(\begin{array}{c} a_{X}\\ a_{Y}\\ a_{Z} \end{array}\right),$$

where $a_{X}$, $a_{Y}\;$and $a_{Z}$ are the components of the tag acceleration measured along the X, Y and Z axes and α is a random rotational angle, as per several authors [12,18,26]. Every classification window was rotated by a random angle.

To balance data for all behavioral classes, data windows from the minor classes were randomly taken, rotational augmentation on a random angle was performed and the result was added to the training set. The balancing was performed for data collected from each individual cow for one day. An increase in the dataset size due to the balancing depended on the level of imbalance and was, on average, 41 ± 20%.

Postprocessing of the classified behavior was achieved by a median filter with a window length equal to five. Both the data collected for this study and the data published by [10] were processed using the same procedure. Additionally, the sampling frequency of the Pavlovic [10] dataset was changed, using zero padding, from the original 10 Hz to the 25 Hz used in the current system.

### 2.5. Tested Classifying Models

**Figure 4:**
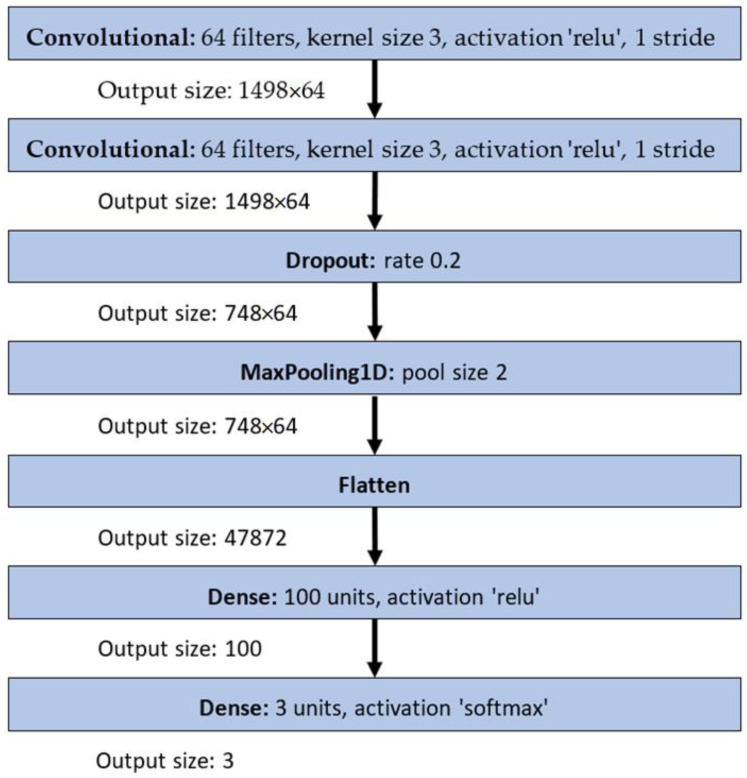
CNN2 architecture.

Two NN classifiers found in the reviewed literature were compared in this study: 

CNN2. Methods for the human activity recognition, described in [27]. The CNN2 consists of two 1D convolutional layers with a kernel size of 3, a dropout layer and a pooling layer, as presented in Figure 4. 

CNN4. Deep CNN for cow activity recognition, as described in [10]. The CNN4 consists of four 1D convolutional layers with kernel sizes of 52 and 1, a dropout layer and a pooling layer.

The models’ structures are available in the Supplementary Materials https://github.com/cowbhave/CowBhave_BehaviorModel (accessed on 2 January 2023).

The size of the classification window was optimized similarly to what found in the reviewed studies [28,29]. In this study, the optimal classification window size was searched by a grid search algorithm in the set [5 10 30 60 90 120 180 300] s with the extreme values 5 s and 300 s, which were taken from Table A1 for cows. The total amount of the bouts for each behavior class in the available labeled dataset with a length less than 300 s was 3.1% for feeding, 2.7% for rumination and 9% for the other behaviors. 

The pretrained classification models for the transfer learning for different window sizes were trained by the data published by [10]. The transfer learning was achieved by training the last convolutional layer in the models (the second for the CNN2 and the fourth for the CNN4 model) and all subsequent layers. The training was performed for 30 epochs with a 0.001 learning rate. An Adam optimizer was used. The model was implemented in Python and trained using the Keras library with Tensorflow.

The datasets used for training (which were the dataset collected in this study and the dataset published by [10]) were acquired from different cows and environments with different sensors, sampling rates and rates of missing data. Hence, they were used to test the applicability of the trained models for other cows and environments. The classification accuracy of models trained on one dataset and validated by another dataset was estimated.

### 2.6. Analysis on the Effect of Training Dataset Size

To evaluate the dependence of the model accuracy on the amount of data used for the model training, learning using randomly initialized model weights and transfer learning were performed using different parts of the original dataset. The smallest data amount which can be used for training is the classification window. For this test, only a 60 s window, including 60 × 25 = 1500 acceleration samples (for which 25 Hz was the accelerometer sampling frequency), was used. Each window had its behavior class label; hence, it was defined as a training sample and the dataset size was measured by the number of training samples. Small testing datasets were created as parts of the original dataset. The original acceleration data for one cow for one day was stored in one file, resulting in a total of 56 files. To create the minimal dataset, one training sample for all three classes was taken from each file, totaling 3 × 56 = 168 training samples (3 × 56 × 60 s = 10,080 s = 2.8 h). This amount represents 0.34% of the total dataset size of 48,540 training samples. For the next dataset, including two training samples taken from each file, the dataset size was 3 × 56 × 2 = 336 training samples. Hence, in the analysis, the initial datasets, including the following dataset sizes, were used: 168, 336…1680, 4854, 9708…48,540. In larger datasets for which an equal amount of training samples for each behavior class could not be taken, actual data size for the training was increased by resampling. The sizes of the corresponding datasets enlarged by the augmentation and balancing were: 336, 504…3192, 13,878, 28,358…132,762. For each fold in the 10-fold validation, the size of dataset was multiplied by a factor equal to approximately 9/10.

### 2.7. Accuracy Evaluation

The accuracy was estimated by the total classification precision and macro F1, confusion matrix, and (micro) $Precision_{i}$, $Recall_{i}$ and $F1_{i}$ for each separate behavior class, *i,* as follows in Equation (2):

(2) $$Precision_{i}=\frac{TP_{i}}{TP_{i}+FP_{i}},\;Recall_{i}=\frac{TP_{i}}{TP_{i}+FN_{i}},\;F1_{i}=2\frac{Precision_{i}\cdot Recall_{i}}{Precision_{i}+Recall_{i}},\;i=1,2,3;\;Precision=\frac{TP}{TP+FP},F1=mean{\left(F1_{i}\right)}_{i=1,2,3},$$

where $TP_{i}$ is the number of true positive classifications for the class *i*, $FP_{i}\;$is the number of false positive classifications for the class *i*, and $FN_{i}$ is the number of false negative classifications for the class *i*. *TP* is the total number of correct classifications for all classes and *FP* is the total number of incorrect classifications for all classes.

## 3. Results

**Figure 5:**
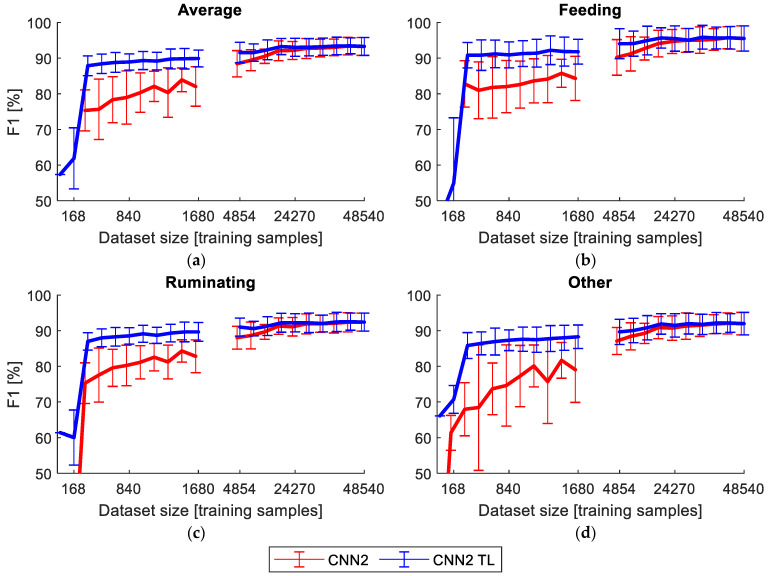
F1 score of the model CNN2 trained using randomly initialized model weights (CNN2) and by the transfer learning (CNN2 TL) for the window sizes of 60 s, depending on the training dataset size measured in training samples taken from the original dataset for the average F1 (**a**), feeding (**b**), rumination (**c**) and other behavior (**d**). The corresponding actual data after augmentation and balancing are: 336,…;3192, 13,878,…;132,762. The error bars represent the STD for 10-fold validation.

The performance of the CNN2 model for the 60 s window size, trained using random weights and by transfer learning for different dataset sizes used for the training, is presented in Figure 5. The use of transfer learning clearly improved the performance (F1 score) of the classifier for small dataset sizes, and the average F1-score 87% was obtained with just 336 training samples. The advantage of using transfer learning disappeared when over 24,000 training samples were used.

**Figure 6:**
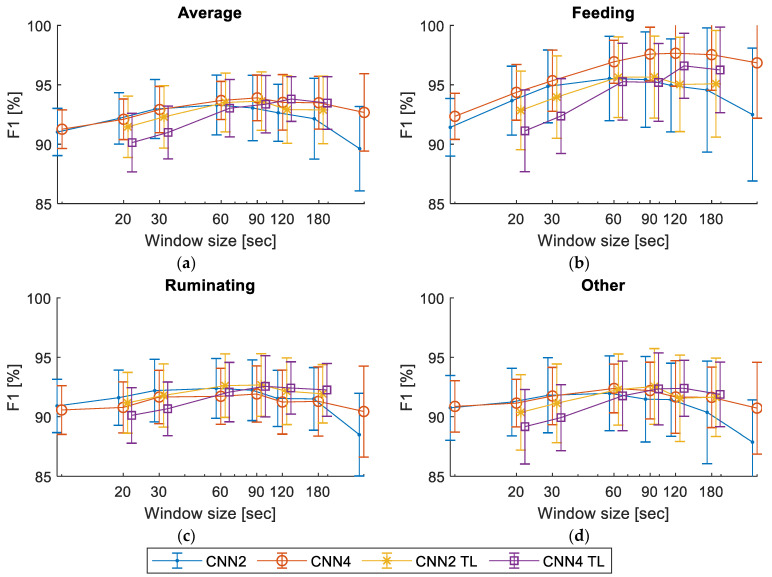
Performance of the tested models, CNN2 and CNN4, trained using randomly initialized model weights and by transfer learning (CNN2 TL and CNN4 TL), depending on the window size for the average F1 (**a**), feeding (**b**), rumination (**c**) and other behavior (**d**). The error bars represent the STD for 10-fold validation.

The training window size had a clear effect on the model performance when training the models on the whole original dataset. The F1 scores for the classifiers CNN2 and CNN4, trained using randomly initialized model weights and by the transfer learning for all tested window sizes, are presented in Figure 6. The highest F1 scores were obtained for the 90 s window size.

The best accuracy scores of all classifiers trained with the full dataset for a window size with the highest average F1 score for each model are presented in Table 2. There were only minor differences in performance between the simpler CNN2 and the more complex CNN4 and between using transfer learning and training using randomly initialized model weights. For all the tested models, the clear optima were in the 60–120 s range.

The accuracy of the models trained on one dataset and validated by another was low: F1 = 57.3% for the model trained by the dataset published by [10] (the pretrained model before transfer learning), and F1 = 63.0% for the model trained by the dataset collected in this study and validated by dataset published by [10].

## 4. Discussion

Using the transfer learning technique, a relatively high (F1 = 87%) behavioral classification accuracy was reached with less than 500 training samples, a significantly better performance (12% higher) compared to the model initialized with random weights. An even higher classification performance was reached by using more training data. In this study, the average F1 score reached its maximum level with around 30,000 training samples, and the use of transfer learning was beneficial up to 24,000 training samples.

The differences in accuracy between the simpler CNN2 and the more complex CNN4 architectures and between the learning model using randomly initialized model weights and transfer learning were not significant when the full dataset was used. This may suggest that increasing the number of layers in an NN does not significantly increase the classifier accuracy for accelerometer data. Additionally, using transfer learning can improve the model performance when large amounts of training data cannot be collected.

The analysis of different training dataset sizes showed a constantly increasing accuracy when more training data was used. However, it also shows the limitation of the classifier, as increasing the dataset size to over 30% did not significantly increase the F1 score from the level of approximately 94%. Nevertheless, this effect occurred in this specific case for which the data were collected with specific sensors in the same environment for the same animals during a short period of time. The influence of the condition diversity on the accuracy of the models should be further studied.

The feeding behavior classification model with the best performance was CNN4, which had an average 93.9% F1 score for the 90 s optimal window size; this is close to the median among the values found in the literature review (Table A1). In practice, classification models are run on continuously measured data that are not split into windows containing data from only one behavior. A large window size can therefore create additional uncertainty in the classification accuracy because sample windows can contain mixed behavioral classes. The F1 score achieved in this study was high compared to the systems using an NN for cow behavior classification, such as those by [10] with F1 = 82%, [11] with F1 = 88.7% and [12] with F1 = 94.4%. Among the systems using machine learning with the performance reviewed in Table A1 were those by [16] with F1 = 93.3%, by [29] with F1= 98.51%, and by [15] with a total accuracy of 98%. An exact comparison of the accuracy scores is impractical due to differences in the research conditions, such as the experimental environment, sensors and the amount and type of the collected data. However, the results in the mentioned studies can be applicable in actual conditions. Having the data openly available would be beneficial for comparison of the developed methods. Data collected in this study and other freely available datasets are listed at https://github.com/Animal-Data-Inventory/PLFDataInventory (accessed on 2 January 2023).

The low accuracy (F1 = 57.3% and F1 = 63.0%) of the models trained and validated by datasets achieved from different cows, different sensors and different reference data and environments showed that both models were not applicable to other environments without additional fitting. However, the high accuracy of the transfer-learned models can mean that the basic patterns of the cow motions characterizing this class of problem were effectively learned by the convolutional layers of the models and used regardless the differences in the sensors and labeling methods.

The development of a machine-learning-based classifier includes a large number of elements and processes; hence, all model parameters could not be chosen optimally. Some of them were manually fine-tuned or adopted from previous studies. Among these parameters are ones related to the data collection (sampling frequency, number of measured axes, variability of animals and environments), data pre-processing (filter parameters, data fixing and augmentation methods) and NN architecture (number, type and size of the layers, number of filters, size of kernels, etc.).

During the experiments, it was found that augmentation simulating the sampling loss implemented by [25] did not improve the performance of the tested models. Due to frequent missing data intervals with a length of 10–20 samples, the data imputations performed by [29,30] were not effective for this system.

The information missing rate during the data transfer from the tags was about 50%, while it decreased when the number of tags was decreased. The main reason for this was connected with the large number of sent data messages, which led to hardware limitations [31]. The number of messages depends on the number of tags fitted to the cows and the sampling frequency, which was set to 25 Hz. However, the number of cows enrolled in this study was typical for a commercial barn, and this kind of measuring system should be able to perform in set conditions. An additional analysis of data redundancy must be conducted to find out to what extent the sampling frequency can be decreased in order to diminish the information loss or to increase the number of cows in the same compartment.

During the development and analysis of the classification model, a large number of assumptions were made to achieve practical results (e.g., the classification model and the estimation of a sufficient amount of the training data) in a realistic environment (e.g., low quality data, different sensors) with limited available data. The analysis of these assumptions must be performed in the future research. However, the results achieved in this study appear promising for practical applications.

In future work, additional uncertainties related to the training of classifiers should be studied. The limits of the applicability of trained models should be tested by application on datasets collected in different conditions and environments. To achieve this aim, additional data collection or the adoption of existing datasets is required. The influence of the data collected from a specific animal [32] on the classification accuracy of the entire herd should be also tested. The minimal amount of data transferred from the sensors should be evaluated by reducing the number of measured acceleration axes and the sampling frequency.

## 5. Conclusions

In this study, we developed a low-cost, open-source system for feeding behavior classification of dairy cows with an average F1 score equal to 93.9% and analyzed different methods and amounts of required training data. A dataset of approximately 20 cow × days for learning using randomly initialized model weights and approximately 10 cow × hours for the transfer learning were sufficient to achieve F1 = 90%. Despite a relatively high classification accuracy, additional research is needed to evaluate the applicability of the classifier to other environments and conditions.

## Figures and Tables

**Table 1:** Characteristics of datasets used for NN model training (mean ± STD).

	*N*	Period, Days	Average Time, Hours	Total Time, Hours (Days)	Fe, %	Ru, %	Oth, %
Collected data	21	1–3	38.5 ± 12.4	809 (33.7)	19.7 ± 5.7	36.9 ± 6.1	43.3 ± 6.9
Open-source data	18	6–18	191.7 ± 87.5	3450.5 (143.7)	17.6 ± 3.8	38.4 ± 3.5	43.9 ± 6.6

*N*—number of cows, Fe—feeding, Ru—ruminating, Oth—other behaviors.

**Table 2:** Comparison of model performance trained by simple training and transfer learning. Precision, F1 and recall values are given averaged (mean ± STD) for a 10-fold validation for optimal window sizes (WS), presented in Figure 6a.

	CNN2	CNN4	CNN2 TL	CNN4 TL
Precision	92.9 ± 2.5	93.3 ± 2.0	93.3 ± 2.5	93.3 ± 1.9
F1	93.3 ± 2.5	93.9 ± 1.9	93.6 ± 2.4	93.8 ± 1.8
Recall	94.2 ± 1.7	94.3 ± 1.5	94.5 ± 2.5	94.4 ± 1.4
WS (s)	60	90	90	120

## Data Availability

Bloch, V.; Frondelius, L.; Arcidiacono, C.; Mancino, M.; Pastell, M. 2022. Data from neck acceleration tags for training cow’s feeding behavior classifier. Zenodo, v1. https://doi.org/10.5281/zenodo.6784671.

## Supplementary Materials

The structure of the CNN classifier models is available in the Supplementary Materials: https://github.com/cowbhave/CowBhave_BehaviorModel (accessed on 2 January 2023).

## Appendix A

To recognize the cow feeding behavior, different machine learning (ML) methods have been used in research studies, as listed in Table A1. A specific set of features was used in each study which was extracted from the acceleration samples in time and frequency domains. However, analyses of the feature types and their amount and physical interpretability have not been found.

**Table A1:** Review of methods for dairy cow behavior recognition based on acceleration sensors. Studies using NN-based methods are highlighted in bold.

Publication	Behavior Types	Interval Length, Sampling Rate	Number of Features	Method	Accuracy (F1)	Animals, Period, Barns
Achour, 2019 [33]	S, L, transition	3–10 s, 1–4 Hz	2	DT	99%	8, 0.25, 1
Arcidiacono, 2017 [16]	F, S	5 s, 4 Hz	1	DT	93.3%	5, 5 h, 1
Barwick, 2018 [34]	G, W, S, L	10 s, 12 Hz	12	Quadratic discriminant analysis		5, 2.5 h, 1
Benaissa, 2017 [35]	F, Ru, other activity	60 s, 10 Hz	9	DT, SVM	94.4%	10, 6 h, 1
Dutta, 2015 [36]	G, searching, W, Ru, Re, scratching	5 s, 10 Hz	9	probabilistic principal components analysis, fuzzy C means, self-organizing map	89%	
**Eerdekens, 2020 (horses)** [37]	S, W, trot, canter, roll, paw, flank watching	2.1 s, 25 Hz		CNN	97.84%	
Kaler, 2019 (sheep) [38]	W, S, L	7 s, 16 Hz	16	RF	80%	18, 1.6
**Li, C., 2021** [12]	F, W, salting, Ru, Re	10 s, 25 Hz		CNN	94.4%	6, 6 h, 1
**Pavlovic, 2021** [10]	F, Ru, Re	10 Hz, 90 s		CNN	82%	18, 6–18 d, 1
Pavlovic, 2022 [39]	F, Ru, Re	10 Hz, 90 s		Hidden Markov model, LDA, partial least squares discriminant analysis	83%	18, 6–18 d, 1
**Peng, 2019** [11]	F, L, Ru, licking salt, moving, social licking and head butt	3.2–12.8 s, 20 Hz		RNN with LSTM, CNN	88.7%	6, ?
Rahman, 2018 [40]	G, S, Re, Ru	200 samples, 12 Hz	6	Majority voting, WEKA		?, ?
Rayas-Amor, 2017 [3]	G, R	30 s	2	Linear regression	96.1(R2)	7, 9
Riaboff, 2020 [15]	G, W, Ru, Re	10 s,		Extreme boosting algorithm, Adaboost, SVM, RF	98% accuracy	86, 57 h, 4
Shen, 2019 [41]	F, Ru, O	256 samples, 5 Hz	30	K-nearest neighbor, SVM, PNN	92.4%	5, ?
Simanungkalit, 2021 [42]	Licking, F, S, L	10 s, 25 Hz	8	DT, RF, KNN, SVM	95–99% accuracy	4, 3.5 d
Tian, 2021 [28]	F, Ru, running, Re, head-shaking, drinking, W	?, 12.5 Hz	9	KNN, RF, KNN-RF fusion	99.34%	20, 3, 1
Vázquez Diosdado, 2015 [43]	F, S, L	300 s, 50 Hz		DT, SVM	91.7%	6, 1.5
Vázquez Diosdado, 2019 (sheep) [44]	W, S, L	7 s, 16 Hz	1	k-means, KNN	60.4%	26, 39
Walton, 2019 (sheep) [45]		5–7 s, 16–32 Hz	44	RF	91–97%	
Wang, 2018 [46]	F, L, S, W	5 s, 1 Hz		Adaptive boosting algorithm	75%	5, 25 h
Wang, 2020 [47]	Estrus	0.5–1.5 h, 1 Hz		KNN, back-propagation neural network, LDA, classification and regression tree	78.6–97.5%	12, 12 d
Williams, 2019 [48]	G, Re and W			13 ML algorithms	93%	40, 0.25

G—grazing; F—feeding; L—lying; O—other behaviors, excluding those already listed; Re—resting; Ru—rumination; S—standing; W—walking; SVM—support vector machine; DT—decision tree; LDA—linear discriminant analysis; KNN—K-nearest neighbor; ? (or empty)—data was not found in the publication.
